# Acute and protracted abstinence from methamphetamine bidirectionally changes intrinsic excitability of indirect pathway spiny projection neurons in the dorsomedial striatum

**DOI:** 10.1038/s41598-022-16272-6

**Published:** 2022-07-15

**Authors:** Sanghoon Choi, Yijuan Du, David L. Wokosin, Steven M. Graves

**Affiliations:** 1 Department of Pharmacology, University of Minnesota, Minneapolis, MN USA; 2 Department of Neuroscience, Feinberg School of Medicine, Northwestern University, Chicago, IL USA

**Keywords:** Neuroscience, Cellular neuroscience, Neuronal physiology, Reward

## Abstract

Methamphetamine (meth) is an addictive psychostimulant and illicit use presents significant personal and socioeconomic harm. Behavioral studies support the involvement of the dorsal striatum in drug-seeking but stimulant induced dysfunction in this region is understudied. The dorsal striatum can be subdivided into the dorsomedial (DMS) and dorsolateral (DLS) striatum with the DMS implicated in goal-directed and DLS in habitual behaviors; both regions are primarily composed of GABAergic direct (dSPNs) and indirect pathway (iSPNs) spiny projection neurons. To examine the effect of repeated meth on SPNs, mice were administered meth (2 mg/kg) for ten consecutive days and intrinsic excitability, dendritic excitability, and spine density were examined. DMS iSPN intrinsic excitability was increased at 1 day but decreased at 21 days of abstinence. In contrast, DMS dSPN intrinsic excitability was unchanged at either timepoint. Dendritic excitability and spine densities were unaltered in DMS iSPNs and dSPNs at 1 and 21 days of abstinence. The effect of repeated meth on iSPN excitability was specific to the DMS; DLS iSPN intrinsic excitability, dendritic excitability, and spine density were unchanged at 1 and 21 days of abstinence. These findings point toward DMS iSPN dysfunction in meth use disorders with differential dysfunction dependent on abstinence duration.

## Introduction

Methamphetamine (meth) is a potent psychostimulant and the number of individuals suffering from a meth use disorder, overdose deaths, and emergency department visits in the US has increased in recent years^1–4^. The primary pharmacological targets of meth are plasmalemmal reuptake proteins, particularly dopamine transporters, and vesicular monoamine transporter 2 (VMAT2)^5,6^. Binding of these transport proteins by meth results in elevated cytosolic and synaptic concentrations of dopamine ultimately resulting in euphorigenic and reinforcing effects. There has been a strong emphasis on understanding the effects of drugs of abuse on the nucleus accumbens and the role this brain region plays in substance use disorders; however, clinical and preclinical evidence indicates that the dorsal striatum is also an important region in need of further study. Imaging data in human subjects provide evidence that the dorsal striatum is involved in stimulant craving and that dopamine levels in this region may even correlate with craving severity^7–11^. Consistent with clinical evidence, dopamine receptor antagonism, inactivation, lesioning, and chemogenetic modulation of neuronal function within the dorsal striatum inhibits stimulant-seeking behavior in rodent models^12–21^.

Despite clinical and preclinical evidence implicating the dorsal striatum in substance use disorders the effects of stimulant administration, and in particular meth, on neuronal function in the dorsal striatum is unclear. The dorsal striatum is primarily composed of GABAergic spiny projection neurons (SPNs). These SPNs can be further subdivided into D1 dopamine receptor expressing direct pathway SPNs (dSPNs) which project to the substantia nigra pars reticulata and internal segment of the globus pallidus and D2 dopamine receptor expressing indirect pathway SPNs (iSPNs) which project to the external segment of the globus pallidus^22^. Additionally, the dorsal striatum can be subdivided into the dorsomedial (DMS) and dorsolateral (DLS) striatum, both of which are innervated by substantia nigra pars compacta (SNc) dopamine neurons. Electrical and optogenetic stimulation of the SNc is rewarding^23–26^ and the DMS in particular is associated with goal-directed behavior whereas the DLS is more heavily involved regulating habit and motor control^27–30^.

The goal of the present study was to investigate the consequence of repeated meth administration on SPN function and anatomy in the dorsal striatum. Mice were administered meth (2 mg/kg) non-contingently for ten consecutive days and SPN intrinsic excitability, dendritic excitability, and spine density were examined after either 1 or 21 days of abstinence. Repeated non-contingent meth administration uniquely altered the intrinsic excitability of iSPNs in the DMS leaving DLS iSPNs unaffected. Additionally, the effect on DMS iSPN excitability was dependent on abstinence duration with an acute 1 day of abstinence producing an increase and protracted 21 days of abstinence a decrease in intrinsic excitability.

## Results

### DMS iSPN but not dSPN intrinsic excitability was increased during acute abstinence from meth

**Figure 1:**
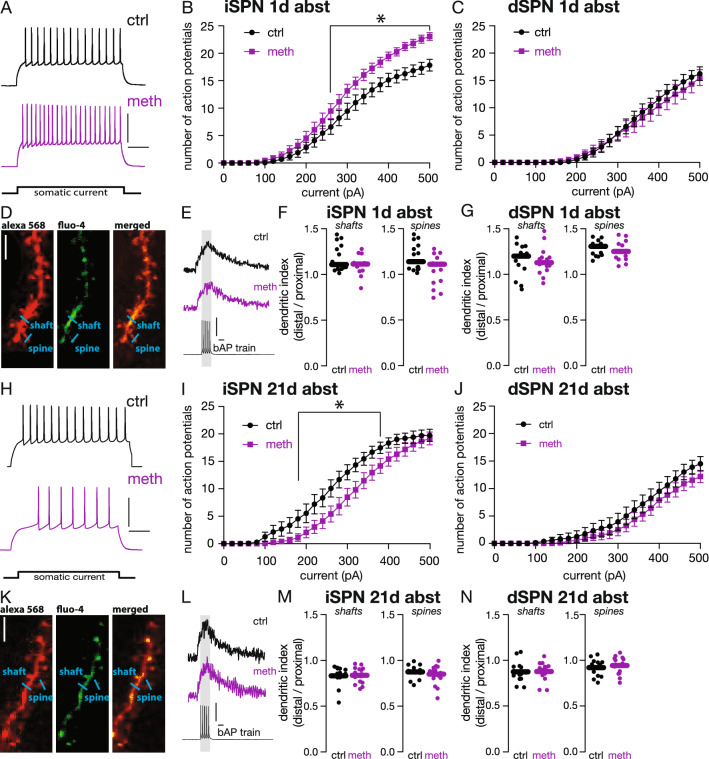
DMS iSPN intrinsic excitability was increased at 1 day and decreased at 21 days of abstinence from repeated methamphetamine administration. (**A**) Sample traces of DMS iSPNs from mice treated with saline (ctrl) and methamphetamine (meth) with 380 pA current injection; horizontal scale bar indicates 100 ms and vertical scale bar indicates 50 mV. (**B**) DMS iSPN intrinsic excitability was increased from 260 to 500 pA after 1 day abstinence (ctrl n = 16 neurons/6 mice; meth n = 19 neurons/7 mice). Two-way ANOVA showed significant effects of injected current (F_(25,450)_ = 308.8, p < 0.0001), treatment (F_(1,18)_ = 7.698, p = 0.0125), and injected current X treatment interaction (F_(25,320)_ = 4.911, p < 0.0001); Fisher’s LSD *post-hoc* analysis determined significant differences at 260–500 pA. (**C**) Repeated meth had no effect on DMS dSPN intrinsic excitability (ctrl n = 17 neurons/7 mice; meth n = 19 neurons/8 mice). Two-way ANOVA showed significant effect of injected current (F_(25,450)_ = 81.88, p < 0.0001) but not treatment (F_(1,18)_ = 0.3971, p = 0.5365) or injected current X treatment interaction (F_(25,398)_ = 0.7483, p = 0.8064). (**D**) Sample images of SPN dendrites with the anatomical dye Alexa 568 (red; left), calcium sensitive dye Fluo-4 (green; middle) and both dyes merged (right). Example regions sampled (spine and shaft) for line-scans using two-photon laser scanning microscopy are indicated with blue lines. (**E**) Sample Fluo-4 fluorescent traces collected during backpropagating action potentials (bAPs) and voltage recording of bAPs (bottom); horizontal scale bar denotes 100 ms and vertical scale bar denotes 25 mV. (**F**) DMS iSPN dendritic excitability was unaffected at 1 day abstinence. Data analyzed using unpaired *t*-tests (shaft: t (33) = 0.1693, p = 0.8666, two tailed; spine: t (34) = 0.1598, p = 0.8739, two-tailed). (**G**) DMS dSPN dendritic excitability also was unaffected at 1 day of abstinence. Data analyzed using unpaired *t*-tests (shaft: t (34) = 0.6704, p = 0.5071, two-tailed; spine: t (34) = 0.2922, p = 0.7719, two-tailed). Dendritic excitability was assessed in the same neurons used to measure intrinsic excitability and sample sizes are identical. (**H**) Sample traces from DMS iSPNs from control and meth treated mice with 380 pA current injection. The horizontal scale bar denotes 100 ms, and vertical scale bar denotes 20 mV. (**I**) DMS iSPN intrinsic excitability was decreased from 180 to 380 pA after 21 days of abstinence from repeated meth administration (ctrl n = 15 neurons/5 mice; meth n = 17 neurons/7 mice). Two-way ANOVA showed significant effects of injected current (F_(25,400)_ = 196.3, p < 0.0001), treatment (F_(1,16)_ = 4.216, p < 0.05), and significant current X treatment interaction (F_(25,348)_ = 2.515, p < 0.0001); Fisher’s LSD *post-hoc* analysis determined significant differences at 180–380 pA. (**J**) DMS dSPN intrinsic excitability was unaffected after 21 days of abstinence from repeated meth (ctrl n = 16 neurons/6 mice, meth n = 16 neurons/6 mice). Two-way ANOVA showed significant effect of current (F_(25,375)_ = 84.87, p < 0.0001) but not treatment (F_(1,15)_ = 0.9763, p = 0.3388) or current X treatment interaction (F_(25,375)_ = 0.6676, p = 0.8880). (**K**) Sample images of SPN dendrites with the anatomical dye Alexa 568 (red; left), calcium sensitive dye Fluo-4 (green; middle) and both dyes merged (right). Example regions sampled (spine and shaft) for line-scans using two-photon laser scanning microscopy are indicated with blue lines. (**L**) Sample Fluo-4 fluorescent traces collected by evoking backpropagating action potentials (bAPs) and voltage recording of bAPs (bottom); horizontal scale bar denotes 100 ms and vertical scale bar indicates 25 mV. (**M**) DMS iSPN dendritic excitability was unaffected at 21 days of abstinence from repeated meth administration. Data analyzed using unpaired *t*- or Mann–Whitney tests as appropriate (shaft: t (27) = 0.1372, p = 0.8919, two-tailed; spine: U = 83, p = 0.4187, two-tailed). (**N**) DMS dSPN dendritic excitability also was unaffected after 21 days of abstinence. Data analyzed using unpaired *t*-tests (shaft: t (28) = 0.0689, p = 0.9455, two-tailed; spine: t (27) = 0.0603, p = 0.9524, two-tailed). Dendritic excitability was measured in the same neurons used to measure intrinsic excitability and sample sizes are identical; *p < 0.05 comparing control *vs.* meth.

Mice were non-contingently administered 2 mg/kg meth in the home cage for ten consecutive days and sacrificed after one day of abstinence (acute abstinence). This dose of meth was selected based on it being a behaviorally relevant dose capable of inducing motor sensitization and conditioned place preference in rodents^31–34^. Additionally, this dose is comparable to amounts self-administered by both rats and mice in short access self-administration paradigms^35–41^. Ex vivo brain slices entailing the DMS were prepared for electrophysiological study using whole-cell patch clamp techniques; iSPNs and dSPNs were identified based on green or red fluorescence, respectively. Patched neurons were subjected to successive sweeps of current injection up to 500 pA with 20 pA steps to generate current-spike response curves. Repeated non-contingent meth administration increased intrinsic excitability of DMS iSPNs resulting in more action potentials generated in response to depolarizing current injection compared to DMS iSPNs recorded from mice administered saline (Fig. 1A,B). This hyperexcitability was significant from 260 to 500 pA current injection with an associated decrease in rheobase (minimum current injection needed to evoke an action potential; Table 1).

**Table 1:** DMS iSPN membrane properties: 1 day of abstinence.

10-day treatment	Saline	Meth
AP Threshold (mV)	−36.41 $\pm$ 1.29	−38.83 $\pm$ 1.70
Rheobase (pA)	217.5 $\pm$ 17.31	190.5 $\pm$ 13.1*
I_R_ (MW)	70.35 $\pm$ 0.64	70.89 $\pm$ 0.98
RMP (mV)	−82.13 $\pm$ 0.81	−80.48 $\pm$ 1.04
AHP amplitude (mV)	13.79 $\pm$ 0.98	11.49 $\pm$ 0.44*
AP height (mV)	62.74 $\pm$ 2.47	63.88 $\pm$ 3.92

Data presented as mean $\pm$ s.e.m. and analyzed using unpaired *t*- or Mann–Whitney tests where appropriate; saline n = 16 neurons/6 mice; meth n = 19 neurons/7 mice; *p < 0.05.

Passive membrane properties including the resting membrane potential (RMP) and input resistance (I_R_) were recorded; active membrane properties including the action potential (AP) threshold, AP height and afterhyperpolarization (AHP) amplitude were calculated using the first action potential at rheobase. Meth-induced DMS iSPN hyperexcitability was associated with a decreased AHP amplitude with no detectable effect on AP threshold, AP height, RMP, or I_R_ (Table 1). Given that only the AHP amplitude was decreased these results suggest decreased calcium-activated potassium channel expression and/or function as a possible mechanism underlying the hyperexcitability. In contrast to the effect of repeated meth on DMS iSPNs, there was no effect on DMS dSPN excitability (Fig. 1C).

### DMS SPN dendritic excitability was unchanged during acute abstinence from meth

To examine changes in dendritic excitability during acute abstinence from repeated meth administration the calcium sensitive dye Fluo-4 (200 µM) and the anatomical dye Alexa 568 (50 µM) were included in the internal recording solution (Fig. 1D). Back propagating action potentials (bAPs) were evoked by somatic current injection and line scans were acquired at proximal and distal dendritic shafts and spines using two-photon laser scanning microscopy^42^ (Fig. 1D,E). Unlike the effects on intrinsic excitability, repeated meth administration had no effect on dendritic excitability in either DMS iSPNs or dSPNs (Fig. 1F,G); bAP data are presented as the dendritic index which is the ratio of distal/proximal fluorescence^42,43^.

### DMS iSPN but not dSPN intrinsic excitability was decreased during protracted abstinence from repeated meth

To determine whether the observed meth-induced change in DMS iSPN intrinsic excitability persisted, mice were treated with saline or meth for ten days after which they underwent a protracted period of abstinence (21 days) in the home cage prior to sacrifice. In contrast to observations during acute abstinence, DMS iSPN intrinsic excitability was decreased after protracted abstinence resulting in fewer action potentials generated in response to 180–380 pA current injection compared to mice treated with saline (Fig. 1H,I). This DMS iSPN hypofunction was associated with a hyperpolarized RMP, a more depolarized AP threshold, increased I_R_, and increased rheobase (Table 2). While the hyperexcitability observed during acute abstinence suggested alteration in calcium-activated potassium channels, findings during protracted abstinence suggest changes in sodium and potassium channel function and/or expression. Unlike the DMS iSPNs however, DMS dSPN excitability remained unaltered (Fig. 1J). To determine whether dendritic excitability was impacted by protracted abstinence, two-photon imaging of calcium influx evoked by bAPs was examined. Consistent with findings during acute abstinence, DMS SPN dendritic excitability was also unaltered during protracted abstinence (Fig. 1K-N).

**Table 2:** DMS iSPN membrane properties: 21 days of abstinence.

10-day treatment	Saline	Meth
AP Threshold (mV)	−42.15 $\pm$ 0.90	−38.32 $\pm$ 1.53*
Rheobase (pA)	181.3 $\pm$ 17.3	232.9 $\pm$ 14.3*
I_R_ (MW)	70.70 $\pm$ 0.86	74.37 $\pm$ 1.1*
RMP (mV)	−83.41 $\pm$ 1.12	−87.65 $\pm$ 1.51*
AHP amplitude (mV)	12.3 $\pm$ 0.66	12.77 $\pm$ 0.70
AP height (mV)	70.76 $\pm$ 2.68	64.36 $\pm$ 3.28

Data presented as mean $\pm$ s.e.m. and analyzed using unpaired *t*- or Mann–Whitney tests where appropriate; saline n = 15 neurons/5 mice; meth n = 17 neurons/7 mice; *p < 0.05.

### DMS SPN spine density was unchanged after acute and protracted abstinence from repeated meth

**Figure 2:**
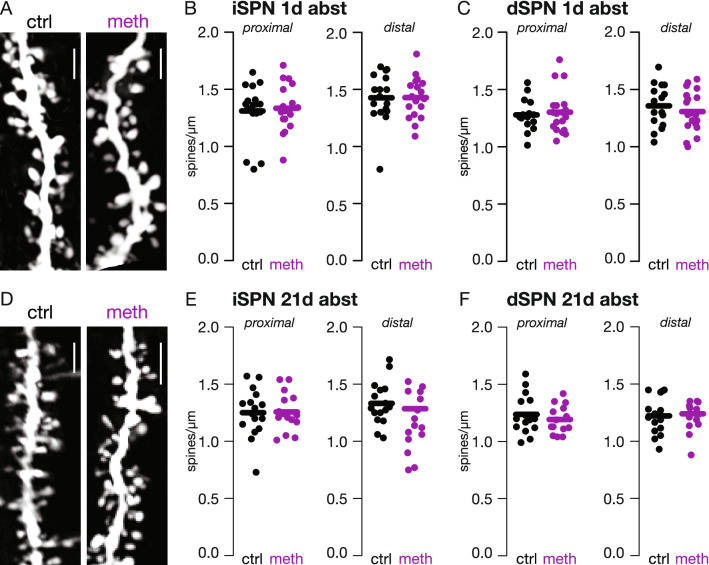
Repeated meth administration had no effect on DMS iSPN or dSPN spine density. (**A**) Sample images of DMS dSPN proximal dendrites from control (ctrl) and meth treated mice after 1 day of abstinence; scale bar denotes 10 µm. (**B**) DMS iSPN spine density was unchanged when measured during acute (1 day) abstinence from repeated meth (ctrl n = 16 neurons/6 mice; meth n = 19 neurons/7 mice). Data analyzed using unpaired *t*- or Mann–Whitney test as appropriate (proximal U = 119, p = 0.5737, two-tailed; distal: t (31) = 0.2832, p = 0.7789, two-tailed). (**C**) dSPN spine density was unchanged at acute (1 day) abstinence from repeated meth (ctrl n = 17 neurons/7 mice; meth n = 19 neurons/8 mice). Data analyzed using unpaired *t*- or Mann–Whitney as appropriate (proximal U = 239.5, p = 0.7824, two-tailed; distal t (34) = 0.6589, p = 0.5144, two-tailed). (**D**) Sample images of DMS dSPN proximal dendrites from repeated control and meth treated mice after protracted (21 days) abstinence. (**E**) DMS iSPN spine density was unchanged after protracted abstinence from repeated meth (ctrl n = 15 neurons/5 mice, meth n = 17 neurons/7 mice). Data was analyzed using unpaired *t*- or Mann–Whitney tests as appropriate (proximal t (29) = 0.6312, p = 0.5329, two-tailed; distal U = 113, p = 0.8219, two-tailed). (**F**) dSPN spine density was unchanged after protracted abstinence from repeated meth (ctrl n = 16 neurons/6 mice, meth n = 16 neurons/ 6 mice). Data analyzed using unpaired *t*-tests (proximal t (27) = 1.312, p = 0.2004, two-tailed; distal t (27) = 1.413, p = 0.1691, two-tailed).

Dendritic spines on SPNs are the site of glutamatergic axospinous synapses that contribute to excitatory drive in otherwise quiescent neurons. The majority of these spines are comprised of corticostriatal circuitry with the remaining axospinous synapses receiving thalamic input, particularly from centrolateral nucleus whereas input from the parafasicular nucleus predominantly forms axodendritic synapses^44–47^. As a first step towards investigating potential circuit dynamics, *z*-stacks were acquired using two-photon laser scanning microscopy to image proximal and distal dendritic segments of DMS iSPNs and dSPNs. Surprisingly spine densities were unaltered by repeated meth administration (Fig. 2). Although these data suggest that the number of axospinous synapses remain unaltered, future studies will be necessary to determine whether functional changes occur such as changes in synaptic strength and/or presynaptic glutamate release.

### Meth effects were specific to DMS and did not occur in DLS iSPNs

**Figure 3:**
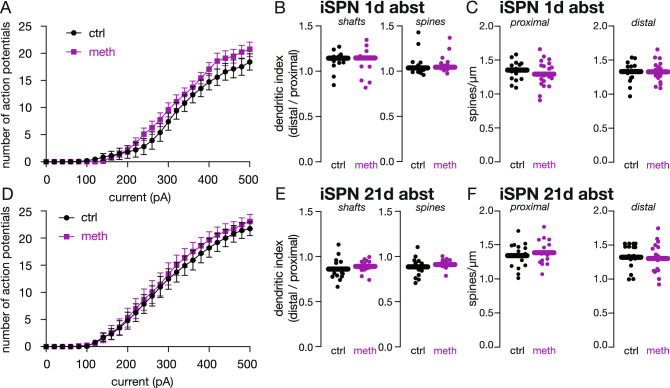
Repeated meth administration had no effect on DLS iSPN excitability or spine density after acute or protracted abstinence. (**A**) Repeated meth had no effect on DLS iSPN intrinsic excitability when measured during acute (1 day) abstinence (ctrl n = 15 neurons/5 mice, meth n = 21 neurons/11 mice). Two-way ANOVA showed significant effect of current (F_(25,400)_ = 210.2, p < 0.0001) but not treatment (F_(1,16)_ = 0.0323, p = 0.8596) or current X treatment interaction (F_(25,348)_ = 0.5678, p = 0.9549). (**B**) Repeated meth had no effect on DLS iSPN dendritic excitability when measured during acute abstinence; sample sizes are identical with those in Fig. 3A. Data analyzed using unpaired *t*- or Mann–Whitney tests as appropriate (shaft t (31) = 1.203, p = 0.2379, two-tailed; spine U = 126.5, p = 0.9629, two-tailed). (**C**) DLS iSPN spine density was unchanged after 1 day abstinence (ctrl n = 15 neurons/5 mice, meth n = 17 neurons/11 mice). Data analyzed using unpaired *t*-tests (proximal: t (31) = 0.5801, p = 0.5661, two-tailed; distal: t (30) = 0.3909, p = 0.6986, two-tailed). (**D**) DLS iSPN intrinsic excitability was unchanged after protracted (21 day) abstinence from repeated meth (ctrl n = 15 neurons/6 mice, meth n = 15 neurons/5 mice). The two-way ANOVA showed significant effect of current (F_(25,350)_ = 186.4, p < 0.0001) but not treatment (F_(1,14)_ = 0.1897, p = 0.6698) or current X treatment interaction (F_(25,350)_ = 0.2308, p > 0.9999). (**E**) Meth had no effect on DLS iSPN dendritic excitability after protracted abstinence; sample sizes are identical to Fig. 3D. Data analyzed using unpaired *t*-tests (shaft: t (25) = 0.7649, p = 0.4515, two-tailed; spine: t (25) = 1.135, p = 0.2670, two-tailed). (**F**) Protracted abstinence from repeated meth did not alter DLS iSPN spine density (ctrl n = 16 neurons/6 mice, meth n = 15 neurons/5 mice). Data analyzed using unpaired *t*-tests (proximal: t (29) = 0.0975, p = 0.9230, two-tailed; distal: t (29) = 0.6713, 0.5073, two-tailed).

Like the DMS, the DLS is also innervated by SNc dopamine neurons and would be pharmacologically targeted by meth. We therefore examined whether the observed changes in DMS iSPN intrinsic excitability extended to DLS iSPNs; mice underwent repeated meth or saline administration and saggital ex vivo brain slices entailing the DLS were prepared after 1 or 21 days of abstinence in the home cage. Unlike the DMS, repeated meth administration had no detectable effect on DLS iSPN excitability when measured after 1 or 21 days of abstinence (Fig. 3A,D). Dendritic excitability and spine density were similarly unaffected (Fig. 3B–F). This presents an interesting dichotomy between the striatal subdivisions which are both innervated by SNc dopamine neurons; however, it is not altogether surprising given that the DLS is a key structure regulating habitual behaviors^29,48^. Given that presented experiments were conducted using non-contingent home cage injections, subjects did not learn to engage in an operant response to gain access to meth, did not develop an association between consumption and explicit cues, or undergo pavlovian-to-instrumental transfer; it is possible that operant paradigms and/or more prolonged duration of meth exposure may be necessary to engage the DLS and induce functional changes.

## Discussion

Presented work demonstrates that repeated administration of meth produced changes in DMS iSPN intrinsic excitability that did not extend to the DLS. Additionally, meth effects were selective for DMS iSPNs leaving dSPN excitability unaltered. Changes in DMS iSPN intrinsic excitability were dependent on duration of abstinence with acute and protracted abstinence producing increased and decreased excitability, respectively. Although further study is necessary to examine synaptic function, the number of axospinous synapses appear to remain constant as repeated non-contingent meth had no detectable effect on spine density in any SPN population examined. Taken together these studies provide a framework to begin to better understand the effects of repeated meth administration on dorsal striatal function with an emphasis on DMS iSPN dysfunction.

During acute (1 day) abstinence DMS iSPN intrinsic excitability was increased with an associated decrease in the AHP amplitude. In contrast to the current findings, repeated cocaine produces hypoexcitability in unidentified accumbal SPNs when measured at 3–4 days of abstinence^49–53^. Unidentified SPNs in the accumbens shell from rats trained to self-administer meth also exhibit hypoexcitability when measured after 1–4 days abstinence^35^. In the current report the observed hyperexcitability did not persist; cocaine produces similar transient changes in striatal immediate early gene expression including FosB and DeltaFosB that dissipates with abstinence^54^. The behavioral implication of this transient change in DMS iSPN excitability is unclear given that this is primarily a characterization study wherein subjects were non-contingently administered meth in the home cage. Nonetheless, optogenetic activation of DMS iSPNs induces punishment^55^, extrapolating from this, DMS iSPN hyperexcitability could potentially contribute to negative affect during early abstinence with an imbalanced DMS favoring iSPN dominance; further study is necessary to definitively determine the behavioral impact of meth-induced DMS iSPN hyperexcitability. In addition, whether this meth-induced hyperexcitability is resultant from repeated meth administration is unclear. In the current report, mice were non-contingently administered meth for 10 consecutive days. One interpretation is that repeated exposure to meth resulted in the observed hyperexcitability; alternatively, the hyperexcitability could be related only to the last injection of meth suggesting that acute exposure to meth may result in hyperexcitability of iSPNs. Additional study will be necessary to determine the duration of meth administration needed to elicit membrane plasticity. The overall duration of this membrane plasticity also deserves future study. The current study shows that DMS iSPN hyperexcitability was transient and no longer present at 21 days of abstinence. Whether this hyperexcitability persists beyond a single day of abstinence is unknown.

The observed hyperexcitability may be resultant from homeostatic membrane plasticity. Accumbal SPNs modify membrane excitability in response to shifts in excitatory synaptic input wherein increasing and antagonizing NMDA receptor activity produces a compensatory shift resulting in decreased and increased membrane excitability, respectively^56^. Meth administration increases synaptic dopamine which inhibits iSPN activity via D2 receptor stimulation and would also favor LTD^57–59^; as a result, iSPN membrane excitability may compensate by increasing excitability in an attempt to restore firing rates. Consistent with this framework, our reported DMS iSPN hyperexcitability was associated with a decreased AHP amplitude; the AHP amplitude is regulated by calcium-activated potassium channels and accumbal homeostatic membrane plasticity was resultant from changes in SK-type calcium-activated potassium channels^56^.

In contrast to acute abstinence, protracted 21-day abstinence resulted in DMS iSPN hypoexcitability. This is consistent with findings in unidentified accumbens SPNs with cocaine, meth self-administration, and cocaine self-administration at 1–4 days abstinence as well as in meth sensitized rats at 17–25 days of abstinence^49–53,60,61^. The observed DMS iSPN hypoexcitability was associated with a more depolarized AP threshold and hyperpolarized RMP but increased I_R_. While the change in the RMP and the change in I_R_ broadly suggest changes in potassium channel expression and/or function, the effect on AP threshold points towards alterations in voltage-activated sodium channels. In the nucleus accumbens, repeated cocaine administration, which produces similar hypoexcitability (measured 3–4 days of abstinence), also causes a more depolarized AP threshold with reduced sodium currents and a hyperpolarized RMP ^49–51^. Additionally accumbal hypofunction is accompanied by decreased high voltage calcium channel potentials^50^. Taken together, current results and existing literature suggest multiple channels may contribute to the observed DMS iSPN hypoexcitability. These findings also suggest a dSPN biased DMS given that dSPN excitability was unchanged while iSPN excitability was reduced. The current report was not designed to investigate behavioral outputs; nonetheless, iSPN hypoexcitabillity without a concomitant change in dSPN function would render a dPSN biased DMS which may contribute to drug-seeking and/or consumption. Consistent with this, optogenetic stimulation of DMS dSPNs is reinforcing^55^ whereas D1 antagonism in the DMS decreases meth self-administration^62^ and meth-seeking^12^. D2 antagonism similarly attenuates meth-seeking behavior^12^ and infusion of the non-specific dopamine receptor antagonist $\alpha$-flupenthixol into the posterior DMS attenuates goal-directed cocaine-seeking^14^. These combined studies suggest that addressing potential imbalance between iSPN and dSPN excitability may mitigate aberrant behaviors associated with stimulant abuse. Based on current findings, D2 antagonism in vivo would directly target meth-induced dysfunction by blocking dopaminergic inhibition of iSPNs, effectively disinhibiting them, whereas D1 antagonism would suppress dopaminergic drive of dSPNs, both of which would theoretically help to restore balance to a dSPN dominant DMS. It is important to re-emphasize that the current report is largely a characterization study. Mice were administered meth in the home cage with the goal of examining the effects of repeated non-contingent meth administration on striatal function. The above potential behavioral implications of the current findings are thus speculative but set a baseline to compare future investigations. Future studies employing self-administration models will be particularly valuable in determining whether (and if so how) contingency further shapes meth-induced striatal plasticity and implications for maladaptive seeking behaviors.

Spines on SPNs are the sites of glutamatergic axospinous synapses with most of these synapses forming corticostriatal circuits and a subset forming thalamostriatal circuits, particularly from centrolateral nucleus^44–47^. In the current report we found no effect of repeated non-contingent meth administration on spine density in iSPNs or dSPNs suggesting that the number of axospinous synapses were unchanged. This is contrasted by a study showing decreased DMS and increased DLS spine density after chronic meth administration^63^. Similarly chronic amphetamine increased DLS spine density^64^. In both aforementioned studies, administration paradigms lasted for upwards of four weeks with an abstinence period of three months or longer. It is unclear whether spine dynamics in the dorsal striatum are resultant from overall lifetime intake of meth/amphetamine, duration of abstinence, or both. Additionally, despite no change in spine density, further study is needed to examine whether synaptic function is altered. For example, both alcohol and nicotine produce increases in DMS NR2B-NMDA currents that, for at least alcohol, contribute to consumption^65–67^.

Overall, the presented studies point towards meth-induced dysfunction in the DMS with an emphasis on iSPNs. Moreover, changes in DMS iSPN excitability was dependent on the duration of abstinence. Further investigation is needed to elucidate the behavioral implications of our findings as it relates to substance abuse, maladaptive seeking behavior, and the potential role of contingency on plasticity differentiating DMS and DLS subregions.

## Materials and methods

### Experimental subjects and treatment paradigm

Mice expressing eGFP under the *drd2* and Tdtomato under the *drd1a* receptor regulatory elements were bred in-house and maintained on a C57Bl/6 background. All experimental subjects were adult (> 8 weeks of age) male mice (n = 59 mice) that were hemizygous for transgenes. Experiments were conducted in male mice exclusively because meth-induced sex differences in rodents are reported at behavioral^68–75^, and molecular^73,75,76^ levels and because estradiol increases amphetamine-induced striatal dopamine release^77^. Future studies will be necessary to explore potential meth-induced sex differences in striatal dysfunction. Procedures were approved by the University of Minnesota Animal Care and Use Committee and in compliance with National Institutes of Health Guide for the Care and Use of Laboratory Animals and ARRIVE guidelines. Mice were group-housed on a 12-h light/dark cycle with food and water provided ad libitum*;* all injections and experiments were conducted during the light cycle. At approximately eight weeks of age experimental subjects were administered daily intraperitoneal (i.p.) injections in the home cage of either (+)-methamphetamine (meth; 2 mg/kg) or saline (10 ml/kg) for ten consecutive days. Subjects remined in the home cage for 1 or 21 days of abstinence prior to sacrifice.

### Ex vivo brain slice preparation

Mice were terminally anesthetized 1 or 21 days after the last saline or meth injection by 50 mg/kg ketamine/4.5 mg/kg xylazine (i.p.) and transcardially perfused with ice cold modified (low calcium) artificial cerebrospinal fluid (aCSF) containing (in mM) 124.0 NaCl, 3.0 KCl, 1.0 CaCl_2_, 2.0 MgCl_2_, 26.0 NaHCO_3_, 1.0 NaH_2_PO_4_, and 16.66 glucose. Brains were removed and sagittal slices (275 µm thick) entailing the dorsomedial (DMS) or dorsolateral (DLS) striatum sectioned (Leica VT 1200S). Collected brain slices were transferred to a holding chamber containing normal aCSF (in mM 124.0 NaCl, 3.0 KCl, 2.0 CaCl_2_, 2.0 MgCl_2_, 26.0 NaHCO_3_, 1.0 NaH_2_PO_4_, and 16.66 glucose) for > 30 min prior to experimentation. Carbogen (95% O_2_/5% CO_2_) was continuously bubbled throughout; aCSF solutions were pH ~ 7.4 and 310–320 mOsm.

### Electrophysiology and two-photon laser scanning microscopy

Ex vivo brain slices were transferred to a recording chamber maintained at 32–34 degrees Celsius with continuous perfusion of carbogen-bubbled aCSF; iSPNs and dSPNs were identified based on eGFP or TdTomato expression, respectively, using an X-cite 110 LED (Excelitas Technologies) on a two-photon laser scanning microscope system (Bruker) consisting of a Nikon FN-1 microscope and Chameleon Ultra II two-photon laser (Coherent Inc.). Identified SPNs were patched under a Nikon 60X/1.00 W NA lens using thick-walled borosilicate glass pipettes (3–4.5 MΩ resistance) pulled on a P-1000 micropipette puller (Sutter Instruments)*.* Patch pipettes were filled with internal recording solution containing (in mM) 135.0 KMeSO_4_, 5.0 KCl, 0.5 CaCl_2_, 10.0 HEPE-K, 2.0 ATP-Mg, 0.5 GTP-Na, 5.0 Phosphocreatine-Tris, 5.0 Phosphocreatine-Na, 0.1 Spermine, 0.05 Alexa 568, and 0.2 fluo-4; pH and osmolarity were adjusted to 7.25–7.30 and 270–280 mOsm, respectively. Whole-cell recordings were obtained in current clamp using a MultiClamp 700B amplifier (Axon Instruments) and PrairieView software (Bruker) consistent with prior studies^42,44^. Intrinsic excitability was examined in current clamp with successive 500 ms somatic current injections (20 pA steps, 5 s sweep duration acquired 5 s apart). The number of evoked action potentials, input resistance, and resting membrane potential were recorded; active membrane properties including rheobase (i.e. minimum current injection needed to evoke an action potential), afterhyperpolarization amplitude, action potential threshold, and action potential height were also recorded. The afterhyperpolarization amplitude and action potential height were calculated as the voltage difference from the action potential threshold. Input resistance and the resting membrane potential are associated with potassium channels whereas the afterhyperpolarization amplitude is largely driven by calcium-activated potassium channel activity. The action potential threshold is linked to voltage-gated sodium channels whereas the action potential peak is the point in which sodium channels begin to inactivate and voltage-gated potassium channels open to repolarize the cell. Changes in these membrane properties therefore provide information that can guide future directions.

Dendritic excitability was assessed by somatic current injection to evoke back-propagating action potentials (bAPs) and measuring the associated calcium signals via Fluo-4 fluorescence. Fluo-4 fluorescence was measured using 810 nm excitation by a two-photon laser (Chameleon Ultra II, Coherent Inc.) at proximal (30–60 µm from the soma) and distal (> 80 µm from the soma) dendrites and spines^42^. Line-scans (1.8 s duration) of dendritic shafts or spines were acquired in triplicate using a 10 µsec dwell time with 0.15 µm $\times$ 0.15 µm resolution; line-scans were acquired ~ 15 min after acquiring whole-cell configuration to allow sufficient time for the dye to load and equilibrate. After background subtraction, the Fluo-4 calcium signal (green channel) was normalized to the fluorescence intensity of the anatomical dye (Alexa 568; red channel) which is insensitive to changes in calcium or voltage; the area of fluorescence change in distal divided by proximal signals was calculated as the dendritic index to correct for potential differences in dye loading, laser power, and optical path, consistent with prior studies.^42^.

After electrophysiological recordings, *z*-series of proximal (30–80 µm from the soma) and distal dendritic (> 80 µm from the soma) segments were obtained using two-photon laser scanning microscopy (810 nm excitation) with 0.15 µm × 0.15 µm resolution, 0.3 µm *z*-steps, and 10 µs pixel dwell time^42,44^. Image J (NIH, Bethesda, MD) was used to deconvolve *z*-stacks^78^ and semi-automated spine counting performed using Neurolucida 360 software (MBF Bioscience, Williston, VT). Spine densities for proximal and distal dendritic segments were averaged per neuron prior to statistical analysis comparing treatment effects.

### Statistical analysis

Prism (GraphPad Software) was used for all statistical analyses. Current-spike response curves are presented as mean $\pm$ s.e.m. and were analyzed using a two-way ANOVA with Fisher’s LSD *post-hoc* analysis; remaining datasets are presented as individual dot plots with a solid line depicting the mean and were analyzed using unpaired *t*-test or Mann–Whitney non-parametric test as appropriate (datasets tested for normality using the Shapiro–Wilk test). Tables present the mean $\pm$ s.e.m. and data analyzed using unpaired *t*- or Mann–Whitney test as appropriate; $\alpha$= 0.05 for all analyses. Experimental results are presented in the text with detailed statistical outcomes provided in figure legends.

## Data Availability

Data are available from the corresponding author upon reasonable request.
